# A Review on Novel Channel Materials for Particle Image Velocimetry Measurements—Usability of Hydrogels in Cardiovascular Applications

**DOI:** 10.3390/gels8080502

**Published:** 2022-08-12

**Authors:** Christina Maria Winkler, Antonia Isabel Kuhn, Gesine Hentschel, Birgit Glasmacher

**Affiliations:** 1 Institute for Multiphase Processes (IMP), Leibniz University Hannover, 30823 Garbsen, Germany; 2 Lower Saxony Centre for Biomedical Engineering, Implant Research and Development (NIFE), 30625 Hannover, Germany

**Keywords:** PIV channel material, hydrogel composites, IOR matching, optical and mechanical properties, material characterization, cardiovascular application

## Abstract

Particle image velocimetry (PIV) is an optical and contactless measurement method for analyzing fluid blood dynamics in cardiovascular research. The main challenge to visualization investigated in the current research was matching the channel material’s index of refraction (IOR) to that of the fluid. Silicone is typically used as a channel material for these applications, so optical matching cannot be proven. This review considers hydrogel as a new PIV channel material for IOR matching. The advantages of hydrogels are their optical and mechanical properties. Hydrogels swell more than 90 vol% when hydrated in an aqueous solution and have an elastic behavior. This paper aimed to review single, double, and triple networks and nanocomposite hydrogels with suitable optical and mechanical properties to be used as PIV channel material, with a focus on cardiovascular applications. The properties are summarized in seven hydrogel groups: PAMPS, PAA, PVA, PAAm, PEG and PEO, PSA, and PNIPA. The reliability of the optical properties is related to low IORs, which allow higher light transmission. On the other hand, elastic modulus, tensile/compressive stress, and nominal tensile/compressive strain are higher for multiple-cross-linked and nanocomposite hydrogels than single mono-cross-linked gels. This review describes methods for measuring optical and mechanical properties, e.g., refractometry and mechanical testing.

## 1. Introduction

Particle image velocimetry (PIV) is a contactless optical measurement method for analyzing fluid dynamics and capturing velocity information. This method offers various applications in disciplines such as aerodynamics, experimental fluid mechanics, and fundamental turbulence research [1]. Besides typical applications such as the characterization of airflows in aircraft cabins [2] and the determination of flows in hollow cylinders [3] or the slip velocity of macroparticles in turbulent flows [4], it is also possible to use PIV to visualize the fluid flow in blood vessels [5]. Utilizing PIV in cardiovascular applications, the main goal and advantage is validating the results of computer simulations (CSs). Hence, these CSs can be expanded into more complex flow issues.

In 2019, the World Health Organization (WHO) estimated that 17.9 million people died of cardiovascular diseases, constituting 32% of all global deaths [6]. These deaths indicate the high medical need for and the importance of cardiovascular research worldwide. In Germany, coronary heart disease caused 92,809 heart surgeries in 2020 [7]. In about half of the cases, coronary bypass surgery was performed [7]. Besides these bypass surgeries, the number of implanted cardiovascular implants is increasing rapidly. The implantation of a vascular prosthesis and the resulting intervention in the cardiovascular system influence the local hemodynamics of the patient’s cardiovascular circulation, affecting the implant’s clinical success. The underlying effects are not yet fully understood. Current research has examined this problem by combining computer-simulated flow behavior and experimental PIV tests.

A major challenge in experimental investigations is the development of a suitable PIV material channel. For the visualization of local flow near the material wall, the channel must not only be designed in an anatomically and biomechanically accurate model but be perfectly matched to the optical requirements for PIV measurements. Especially for cardiovascular flow simulations, the material hydrogel, with its matching optical and mechanical properties, represents a promising novel PIV channel material.

**Figure 1:**
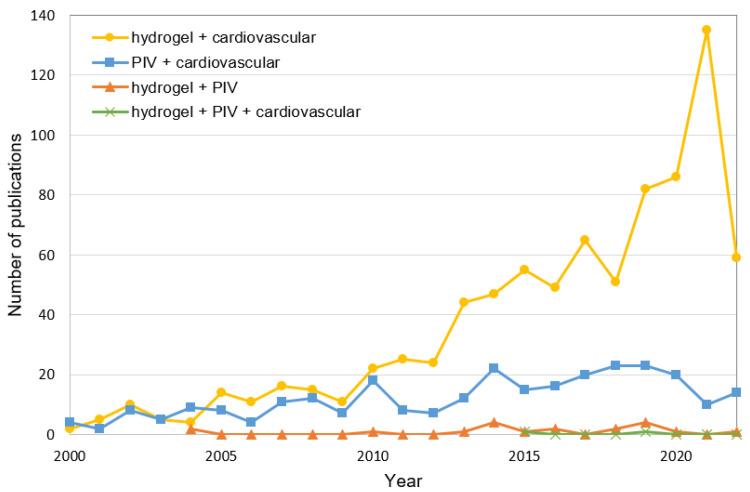
Scopus search: Number of publications in the period 2000–2022 related to the keywords “hydrogel + cardiovascular”, “PIV + cardiovascular”, “hydrogel + PIV”, and “hydrogel + PIV + cardiovascular”.

Performing a general search in Scopus using the keyword “hydrogel”, a total of 89,056 publications were found (period 2000–2022) with an exponentially increasing trend. For the keyword “PIV”, the total number of publications regarding the same period was just 23,286. The total numbers of publications with the keywords “hydrogel + cardiovascular” and “PIV + cardiovascular” were reduced to 837 and 278, as presented in Figure 1. A considerable reduction in publications was seen when the keywords “hydrogel + PIV” were used together. Since 2004, only 19 publications have been listed in Scopus. Finally, with the keywords “hydrogel + PIV + cardiovascular”, a total of two publications were found over the last seven years. A similar trend of publications was determined via the PubMed database.

The Scopus search showed that only a few studies have been performed on hydrogels as a PIV channel material in recent years. Because of the limited information, this review will significantly help researchers who wish to apply PIV in simulations for cardiovascular applications. The review aimed to provide an overview of seven potential hydrogel groups that can be used as PIV channel materials in cardiovascular PIV measurement: poly-2-acrylamido-2-methyl-1-propanesulfonic acid (PAMPS), polyacrylic acid (PAA), polyvinyl alcohol (PVA), polyacrylamide (PAAm), polyethylene glycol (PEG) and -oxide (PEO), sodium polyacrylate (PSA), and poly-N-isopropyl acrylamide (PNIPA). The optical and mechanical properties of these materials are listed in Section 5 based on the optical requirements of PIV channel materials and the mechanical requirements of the physiological and pathological blood vessels to be imaged. Moreover, measurement methods for optical and mechanical properties of hydrogels are presented and discussed.

## 2. Particle Image Velocimetry

**Figure 2:**
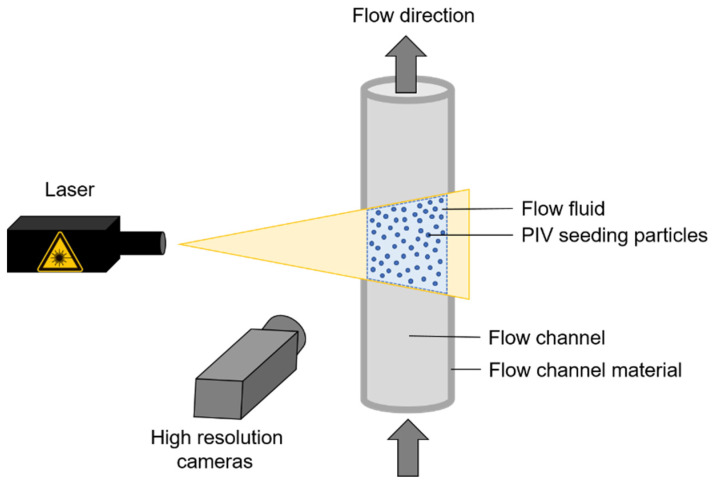
Basic setup of a PIV system with a transparent test area and a laser device for irradiating the PIV particles within the test area. The reflection recording is performed with high-resolution cameras at different time intervals to determine the flow velocities. Adapted from Raffel et al. [1].

For PIV measurements, seeding particles are added to the fluid and temporarily illuminated by a laser. The most commonly used laser type is a solid-state Nd:YAG-Laser with a wavelength λ of 532 nm. Other PIV lasers are the ruby laser, with λ = 694 nm, and the He–Ne laser, with λ = 633 nm [8]. High-resolution cameras record the scattered light of the particles (cf. Figure 2). Images are taken at defined time intervals and used to determine the movement of the seeding particles. Based on this information, it is possible to calculate the average flow velocity, flow direction, and profile [1].

PIV methods are generally divided into 2D- and 3D-PIV. The 2D-PIV method works with two cameras (2C-PIV) in the planar domains of the flow field. With this method, only planar velocity vectors can be determined. By adding a third camera (3C-PIV), 3D-PIV can be performed, and the third velocity vector can be extracted. Standard techniques are the stereo technique, dual-plane PIV, holographic recording, and tomographic PIV [9]. The utilization of different PIV methods depends on the application and the complexity of the fluid flow. For applications in the range of several microns, e.g., blood streams in small vessels, flow fields are determined with µ-PIV systems [9].

### 2.1. Refractive Index Matching

One of the biggest challenges in PIV measurements is matching the refractive index (also known as index of refraction (IOR)) of the solid channel material with that of the fluid [10]. The IOR *n* is a dimensionless material property. It describes the speed at which light travels through a material and is defined as:

*n* = *c*_0_/*c* (1)

where *c*_0_ is the speed of light in vacuum and *c* is the speed of light in phase [11]. The IOR can be measured with a refractometer and is influenced by temperature, material density, fluid, and the laser wavelength of the PIV. For example, the IOR of water is *n* = 1.3333 [12].

**Figure 3:**
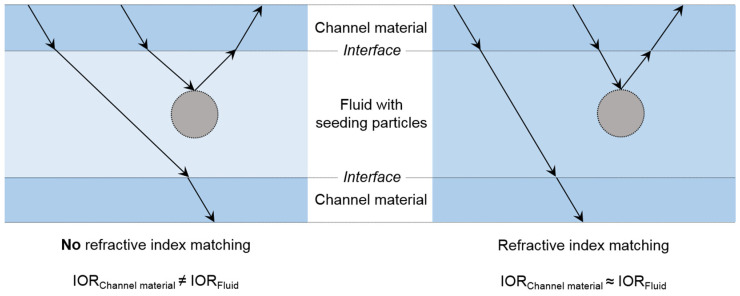
Schematic illustration of refractive index matching between the fluid and channel material: (**left**) the IORs of the channel material and the fluid are not matched, and light rays (arrows) are refracted, which leads to distortions; (**right**) the IORs of the channel material and the fluid are matched, and no refraction of light rays occurs at the interfaces.

IOR matching for PIV is highly relevant, especially for analysis of complex geometries or the local flow near the material wall. Otherwise, seeding particles within the fluid are detected by light distortion, and the results are unreliable for complex geometries [13]. Therefore, the IOR of the fluid is generally matched to that of the channel material. Indeed, the channel material’s transparency is also essential to provide a clear record of particle motion. In Figure 3, IOR matching is schematically drawn for fluids with and without PIV seeding particles.

### 2.2. PIV for Cardiovascular Applications

Via the Scopus search, 278 papers were found focusing on PIV measurements in cardiovascular applications during the last 22 years. PIV measurements can quantify the blood flow conditions near the vessel walls, which makes them very interesting. However, current research has shown limitations in selecting channel materials and IOR matching. Often, rigid materials are chosen that cannot reflect the physiological properties of blood vessels. The fluid’s rheological properties are usually neglected due to IOR matching. The following three main publications on PIV in the cardiovascular field underlined these limitations. Ong et al. [14] evaluated the complex blood flow behavior in the pre- and postaneurismal aorta experimentally by PIV. They developed nonrigid phantom models of the aorta, including an aortic aneurysm, and fabricated them using silicone with an elastic modulus of 2 MPa, close to that of the aorta. The limitation lay in the uniform thickness of the phantom model. To adjust the blood viscosity, a solution mixture of glycerin and water was used with a dynamic viscosity of 3.5 mPa∙s at 23 °C. IOR matching was not implemented [14]. Another study was performed by Stanley et al. [15]. The group produced optically clear anatomical vessel models by 3D printing with a rigid resin. Stanley et al. [15] matched the IOR of the fluid to that of the resin material by utilizing a fluid mixture composed of sodium iodide, glycerol, and distilled water. The IOR of the resin was 1.5304 [15]. The limitations were the stiffness of the model and the postprocess of the inner and outer vessel. Dynamic viscosity was not mentioned. Franzetti et al. [16] also use rigid flow models for PIV measurements on personalized aortic dissection. A potassium thiocyanate (KSCN) water mixture was used as the flow fluid. IOR matching was performed (IOR_Channel material_ = 1.5, IOR_Fluid_ = 1.48). The viscosity of the fluid (2.2 mPa∙s) could not be matched to that of blood, so optical matching could not be performed [16].

Current studies have shown substantial limitations in PIV utilization for cardiovascular applications, as the previously discussed studies by ONG et al. [14], STANLEY et al. [15], and Franzetti et al. [16] underline. For the channel materials, polymethylmethacrylate (PMMA) [17], resin [15], and silicone [14,18,19] have been used. This has made it almost impossible to simulate natural blood vessels. Specifically, the compliance of the vessels has been neglected. Compliance refers to the extensibility of a blood vessel in a radial direction triggered by a physiological pressure load (diastole/systole) [20]. Mixtures of water with glycerin, sodium iodide, and xanthan gum have been used for blood replacement fluids [14,15,18,19,21]. By changing the chemical composition of these solvents, IOR matching can be adapted precisely. However, this consequently changes the blood replacement fluid’s viscosity (blood viscosity is 1.1 mPa∙s at 37 °C [22]). Thus, considering and evaluating the relevance of IOR matching and viscosity is inevitable. The main limitations of rigid channel materials, IOR matching, and viscosity can be neglected by using hydrogels as novel PIV channel materials.

## 3. Hydrogels

In the last two decades, hydrogels have become more and more important. Hydrogels are used in sanitary products such as diapers [23,24], because of their highly absorbent properties, and in the pharmaceutical industry as superdisintegrants [25]. Since the COVID-19 pandemic, hydrogels have also been in particular demand as carriers for disinfectant molecules, allowing them to be used to implement antiseptic coatings with a long-lasting effect against COVID-19 in hospitals [26]. Another popular application area is the biomedical field. Biocompatible hydrogels, in general, are nonthrombogenic [25] and therefore used as scaffolds or contact lenses [27,28]. More precisely, hydrogels are three-dimensional cross-linked polymer networks that are water swollen. In general, hydrogels’ water content is at least 20–30 vol% and can be 90 vol% or more. Chemical or physical bonds hold the polymeric structures of hydrogels together [29]. A unique feature is provided by sensitive hydrogels. These hydrogels change their volume and can therefore respond to external environmental stimuli such as temperature [30], pH value [31], and electric stimuli [32]. If the external stimulus reaches a critical value, volume or shape changes such as swelling, shrinking, or bending can occur [32].

### 3.1. Hydrogel Synthesis

Hydrogels are synthesized via two methods, addition (physical) and condensation (chemical) polymerization [29]. Addition polymerization proceeds as a chain reaction in three stages. The first stage is initiation, leading to the formation of free radicals. An activator-like ultraviolet (UV), gamma, or electron beam starts the chemical reaction, and the initiator opens the monomers’ double bonds. Free radicals react with the monomer and induce a chain reaction [33]. After initiation, propagation follows. This stage includes the linkage of more monomer units to a macromolecule. The process continues until all monomer units have been linked to larger chains. The reaction takes a few minutes, depending on the material and the sample size. The third and final stage of addition polymerization is termination. Reactions can be controlled and ended with termination. This can be done by removing the energy source (e.g., UV light) by directly coupling or exchanging a hydrogen atom from the chains that grow together [33]. The reaction happens without the elimination of side products. Condensation polymerization, the second method, gives the polymer a stepwise growth pattern. It is induced via a condensation reaction or reactions between the functional groups of two polymers [34]. Most approaches use initiators and cross-linking agents such as N,N’-methylenebis acrylamide (MBAm) [35,36,37,38,39,40]. The reaction continues until almost all reagents are depleted. Another difference is the production of side products such as water or carbon dioxide.

### 3.2. Hydrogel Swelling

**Figure 4:**
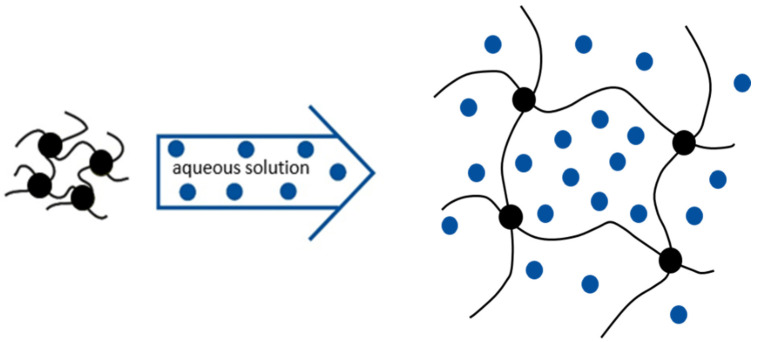
Polymerized hydrogels strongly increase in volume after immersion in aqueous solutions. After completed swelling, hydrogels consist of more than 90 vol% of the working fluid. Adapted from Jorsch [43].

Hydrogels can swell more than 90 vol% when hydrated [41]. The degree of swelling depends on parameters such as temperature, pH value, and ionic strength. Basically, swelling occurs in three phases: 1. diffusion of water into the polymer network, 2. relaxation of the polymer chains by hydration, 3. expansion of the polymer network by relaxation. After fully utilizing the water absorption capacity, the swelling reaches an equilibrium state with maximum liquid loading of the hydrogel. Thus, the osmotic pressure and the elasticity and restoring force of the polymer chains in the network are in equilibrium (cf. Figure 4) [42,43].

### 3.3. Measurement Methods for Optical and Mechanical Hydrogel Properties

The optical and mechanical properties of hydrogels can be measured with different methods. Table 1 lists these properties and the most commonly used measurement methods. A brief description of each method is given, and the relevance of each property to using hydrogels as a PIV channel material is underlined.

### 3.4. Advantages and Disadvantages of Utilizing Hydrogel as a PIV Channel Material

The advantage of using hydrogels as PIV channel materials in the cardiovascular field is their clear optical properties. These are attributable to the fact that hydrogels can absorb up to 90 vol% water. By changing the hydrogel solvents, the transparency and IOR of a hydrogel can be corrected precisely [31]. Thereby, the IOR of the hydrogel channel can be matched perfectly to the index of the used blood replacement fluids. The fluid’s viscosity would be unchanged and would not need to be altered for IOR matching. As already mentioned, the evaluation between IOR and viscosity is thus eliminated. Another advantage of hydrogels is the manufacturing process. Blood vessel models can be cast and adapted to any design, such as complex morphologies for bypasses or ramified blood vessels. Computer tomography (CT) data of real atomic vessels can be implemented. A third advantage of applying hydrogels as blood vessel materials is the imitation of compliance via their elastic properties.

The disadvantages are related to the limited mechanical properties of hydrogels. By swelling, hydrogels absorb large amounts of water, which leads on a molecular level to the liquidlike and solidlike properties of hydrogels [25,41,61]. Therefore, these different properties cause softening and a lack of mechanical strength, which can be measured with tensile and compressive tests (cf. Table 1) [62]. This problem has been solved by synthesizing hydrogels with high mechanical properties, e.g., double or triple networks and nanocomposite gels [61,63].

### 3.5. Double and Triple Networks and Nanocomposite Hydrogels

**Figure 5:**
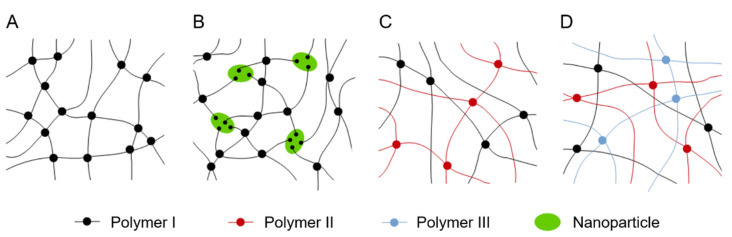
Schematic representation of hydrogel network structures. (**A**) Single network; (**B**) nanocomposite network; (**C**) double network; (**D**) triple network. Adapted from Peak et al. [64].

Double networks are built of two interpenetrating polymer networks (IPNs). The first network consists of highly cross-linked rigid polymers [64]. The second or even third network structure is made of cross-linked flexible polymers, which are looser. Nanocomposite hydrogels are polymerized radically and contain nanoparticles [48,64]. These particles reinforce mechanical stability. Figure 5 visualizes schematically single-, double-, and triple-network hydrogels.

## 4. Hydrogels for PIV Channel Materials

The Scopus search revealed that only two studies performed cardiovascular PIV measurements using hydrogels as a channel material during the last 22 years. Oktamuliani et al. [65] used PIV for visualizing flow velocity vectors based on a left ventricular phantom hydrogel. The phantom hydrogel was produced of PVA with a mixture of dimethyl sulfoxide (DMSO) and water. It possessed transparency and formed a compliant material for pulsatile measurements. The research group also used an aqueous glycerin solution with an IOR of 1.5 as blood substitute [65]. The second study, from Shimizu and Ohta [66], examined changes in flow conditions due to plaque deformation in a stenotic vessel model. Here, transparent PVA hydrogels were also utilized as a channel material. This allowed channel models with different mechanical stiffness to simulate the artery’s changing elasticities due to plaque. As a blood replacement fluid, the group used a working fluid made of glycerol/water solvent and aqueous sodium iodide. The IOR of the fluid was 1.455, and it reduced the optical refraction of the models [66].

Both studies showed that hydrogels are promising PIV channel materials in cardiovascular research. Hydrogels can entirely replace standard materials such as silicon or PMMA. Besides PVA, other hydrogels are presented below that are suitable for cardiovascular PIV simulations. First, the PIV channel material requirements concerning optical and mechanical properties are presented. Then, the selected hydrogel types are briefly described.

### 4.1. Requirements for Optical Properties

IOR matching to the experimental fluid is an essential requirement for a PIV channel material. In current cardiovascular research, typical materials for PIV channels have included PMMA and silicone [17,19]. The IOR of PMMA is 1.491, and that of silicone ranges from 1.40 to 1.44 [17,67]. As blood replacement fluids, mixtures of water with glycerin, sodium iodide, and xanthan gum have been added to water to adjust the fluid’s viscosity to that of human blood [19,21]. The addition of chemicals changes the fluid’s IOR. By comparison, the IOR of water is 1.3325 [35], and that of a glycerin/water mixture is 1.414 [67]. Therefore, the IOR of the hydrogels selected for this review had to be less than 1.55. Another requirement for PIV channel materials is transparency, to enable exact recording of particle movements within the vessel. This review considered only transparent hydrogels with light transmissions of more than 90% [68].

### 4.2. Requirements for Mechanical Properties

The PIV channel, equivalent to a blood vessel, requires elastic materials that mimic blood vessels’ natural compliance. To design suitable PIV channels for the cardiovascular field, mechanical data of physiological and pathological blood vessels are required. For instance, Karimi et al. [69] measured the uniaxial mechanical properties of healthy and atherosclerotic human coronary arteries. The elastic moduli and tensile stresses and strains are listed in Table 2. The values were converted to identical units to compare mechanical properties across the literature in this review. The terminology was adopted by the American Society for Testing and Materials ASTM D638-14 (Young’s modulus equals elastic modulus) [70].

Besides the coronary arteries, many other types of blood vessels are part of the human body. These blood vessels have different properties due to their diameters, wall thicknesses, and degrees of disease. To perform a wide range of physiological and pathological cardiovascular PIV experiments, all mechanical properties (elastic modulus, tensile/compressive stress at break, nominal tensile/compressive strain at break) are addressed in this review. Furthermore, the equilibrium water content of the hydrogel was set to a minimum of 50 wt%. This value was chosen to avoid extreme swelling by the aqueous flow fluid, which would change the hydrogel’s mechanical properties.

In summary, the following ranges for hydrogels were specified for utilization in PIV applications to visualize the blood flow in physiological and pathological vessels:

- IOR: <1.55
- Light transmission: >90%
- Elastic modulus: all values
- Tensile/compressive stress at break: all values
- Nominal tensile/compressive strain at break: all values
- Water content: >50 wt%

### 4.3. Selection of Hydrogels

**Figure 6:**
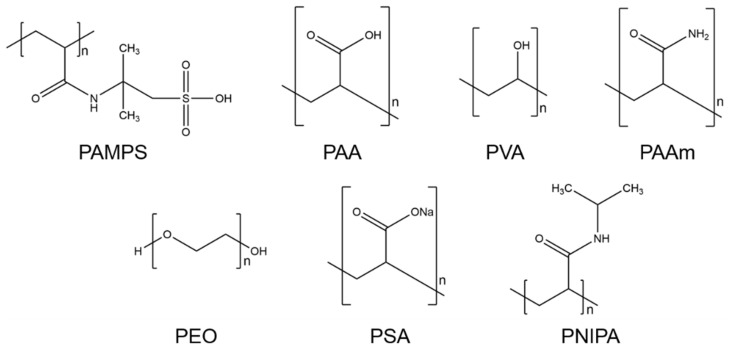
Structural formulae of seven hydrogel groups: poly-2-acrylamido-2-methyl-1-propanesulfonic acid (PAMPS), polyacrylic acid (PAA), polyvinyl alcohol (PVA), polyacrylamide (PAAm), polyethylene glycol (PEG) and -oxide (PEO), sodium polyacrylate (PSA), and poly-N-isopropyl acrylamide (PNIPA).

Considering the set value ranges for optical and mechanical properties, seven hydrogel groups were considered in this review: PAMPS, PAA, PVA, PAAm, PEG and PEO, PSA, and PNIPA. The hydrogels are described briefly with their compositions, main features, and applications. Their structural formulae are pictured in Figure 6.

1. Poly-2-acrylamido-2-methyl-1-propanesulfonic acid (PAMPS)
  PAMPS is a synthetic polymer that consists of acrylic 2-Acrylamido-2-methylpropane sulfonic acid (AMPS). The chemical formula of PAMPS is (C_7_H_13_NO_4_S)_n_. This polymer dissolves well in pure water [71] and is hydrophilic [72]. Furthermore, PAMPS is a thermally stable homopolymer, which induces stability towards thermal degradation [71].
2. Polyacrylic acid (PAA)
  PAA is the polymer of acrylic acid, a compound with the formula (C_3_H_4_O_2_)_n_. PAA exhibits high water retention, and upon absorbing water, it expands over its original size [73]. This hydrophilic property, as well as its propensity as an emulsifying agent, makes it widely marketable. It is commonly used in commercial products for its thickening and suspension properties, e.g., for disposable diapers, adhesives, paints, pharmaceutical drugs, and beauty products [73,74,75,76].
3. Polyvinyl alcohol (PVA)
  The chemical formula of PVA is (C_2_H_4_O)_n_. This polymer is synthetic and highly water soluble. It is produced by the hydrolysis of polyvinyl acetone [73,76,77]. Furthermore, highly polar and hydrophilic solvents can be used to dissolve PVA [73]. This polymer is typically used for rigid and clear optical films, adhesives, and transdermal drug delivery systems. Because of its excellent physical and chemical properties, such as high biocompatibility, low toxicity, and being chemically inert, PVA is broadly used in industrial applications [73,77].
4. Polyacrylamide (PAAm)
  PAAm can be synthesized from the monomer acrylamide by free-radical polymerization [73,76]. The chemical formula is (C_3_H_5_NO)_n_. This polymer can be used as a superabsorbent material. Lightly cross-linked PAAm can absorb and retain large amounts of water and forms a soft gel when saturated [78]. It has other excellent properties for industrial use. For example, PAAm is chemically inert, has low toxicity, and is stable in a wide pH-value range [73,76].
5. Polyethylene glycol (PEG) and polyethylene oxide (PEO)
  PEG with low molecular weight (200 to 20,000 g/mol [79]) is an organic epoxide with the formula (C_2_H_4_O)_n_ [80]. The polymer is known as PEO for higher molecular weights up to 5 million g/mol [79]. Because of its low toxicity, PEG is one of the most used synthetic hydrogels in biomedical applications [73]. PEG polymers are water soluble and can be coupled with hydrophobic molecules to act as surfactants. These polymers are also soluble in methanol, ethanol, benzene, acetonitrile, and dichloromethane [81].
6. Sodium polyacrylate (PSA)
  This cross-linked PSA, with the chemical formula (C_3_H_3_NaO_2_)_n_, is a sodium salt of polyacrylic acid produced by free-radical polymerization [82]. This polymer can absorb a large amount of water because it contains ions, such as carboxyl groups and sodium, in the polymer chain [83]. These give PSA hydrophilic properties that allow it to be classified as a superabsorbent polymer. PSA is widely used in commercial applications, such as cosmetic products, and in general, e.g., in diapers as a thickening agent and in coatings [82,83].
7. Poly-N-isopropyl acrylamide (PNIPA)
  PNIPA is one of the most often utilized temperature-sensitive hydrogels and has the formula (C_6_H_11_NO)_n_ [84]. PNIPA changes its shape by undergoing a discontinuous phase transition at a critical temperature. When this occurs, the polymer chains change from hydrophobic to hydrophilic behavior and make the hydrogel swell. In addition, PNIPA is a biocompatible polymer. Therefore, its applications are found in the biomedical and optical fields [85].

## 5. Review of the Optical and Mechanical Properties of the Selected Hydrogels

The optical and mechanical properties of each hydrogel group are listed in Table 3. Values for both properties were rarely found in one single reference. Optical properties include IOR and light transmission (cf. Section 4.1). For the mechanical properties, the elastic modulus, tensile/compressive stress at break, nominal tensile/compressive strain at break, and water content are listed (cf. Section 4.2). Mechanical properties marked with asterisks (*) were tested under tensile conditions; those not marked were measured under compressive testing methods.

Regarding the optical properties, IOR and light transmission, the single-network hydrogel PNIPA showed the lowest IOR value of 1.32 [53]. The highest IOR value of 1.528 was observed for the hydrogel composition of PAA/polyethylene glycol methacrylate (PEGMA) with a nanotitania hybrid film [50]. The hydrogels PVA with nanocellulose and polyethylene glycol diacrylate (PEG-DA)/methoxy polyethylene glycol (MPEG) had IORs comparable to those of water and glycerin/water mixtures, between 1.333 [44] and 1.4136 [68]. Not every reference contained information regarding the wavelength and temperature for the IOR measurement. Wavelengths ranged from 400 to 650 nm [36,40,47], and temperatures, from 20 to 25 °C [36,40,47]. Furthermore, the double-network hydrogel PEG-DA/MPEG had the highest light transmission of up to 100% [68].

Values for the elastic modulus ranged from 0.0128 MPa [86] to 59.1 MPa [87] for the hydrogel compositions of PAA with sodium silicate and PAAm/polyethylene oxide stat propylene oxide (sPEOPO), respectively. The values for tensile/compressive stress at break ranged from 0.175 MPa* [85] for PNIPAm/polyethylene glycol acrylamide (PEGAAm) to 73.5 MPa for PAMPS/PAAm including silica nanoparticles. Furthermore, PAMPS/polytetrafluorethylene (PTFEA) had the lowest nominal strain at break property of 4.9%* [49]. In comparison, triple-network PSA/PAA/polybutyl acrylate (PBA) showed the highest value of 1730%* [88]. The hydrogels’ water content ranged from 50 to 99.8 wt% [68,89] for PEG-DA/MPEG and PAA with sodium silicate, respectively. Predominantly, hydrogels with water content above ≈85 wt% were found.

## 6. Discussion

This review listed seven different hydrogel groups that fit the optical and mechanical requirements of PIV channel materials for cardiovascular applications, i.e., to perform a wide range of blood flow simulations in physiological and pathological blood vessels. The literature research showed that there are different manufacturing processes for synthesizing hydrogels. For example, the type of polymerization (UV, thermal etc.), the use of solvents, and the concentration of the monomer solutions or swelling time can differ because of changes in optical and mechanical properties. Furthermore, the performances of optical and mechanical measurement methods varied. For example, the wavelength, temperature, and performance under tensile or compressive loading conditions can differ. These deviations made it challenging to compare the properties of each hydrogel. Nevertheless, general relations were observed and compared.

In general, a relation between the IOR and the swelling behavior of the hydrogel was seen. Swelling occurs with increasing water content while the IOR decreases. For example, the water content of the hydrogel PEG-DA/MPEG ranged from 50 to 95 wt%, over which range the IOR decreased from 1.4136 to 1.3388 [68]. Besides the IOR, the transparency increased from 97.6 to 100% [68]. According to different ratios of ionic components in the swelling medium, the degree of hydrogel swelling can differ because of changes in pH values or osmotic pressure [25]; during PIV measurement, the OH– groups of the utilized PIV fluid influenced the swelling behavior, and hence, the mechanical parameters changed as well [25]. Therefore, the swelling of the PIV channel material is an important aspect of experimental PIV setup design.

Another relation existed between the water content and the elastic modulus. With increasing water content, decreases in the elastic modulus was observed. The hydrogel swelling ratio in pure water rose when the total monomer concentration or cross-linking density decreased (e.g., agent MBAm [100]). This led to a reduction in mechanical strength [25,62]. Double-network hydrogels, such as PAMPS/PAAm [37] and PAA/alginate [89] cross-linked with nanoparticles (e.g., silica), showed extraordinary mechanical properties. Under loading conditions, the nanoparticles distributed the applied stress equally to the polymer network and prevented the polymer chains from being destroyed [55,63]. These hydrogels exhibited higher mechanical properties than single- and triple-network hydrogels [49].

**Figure 7:**
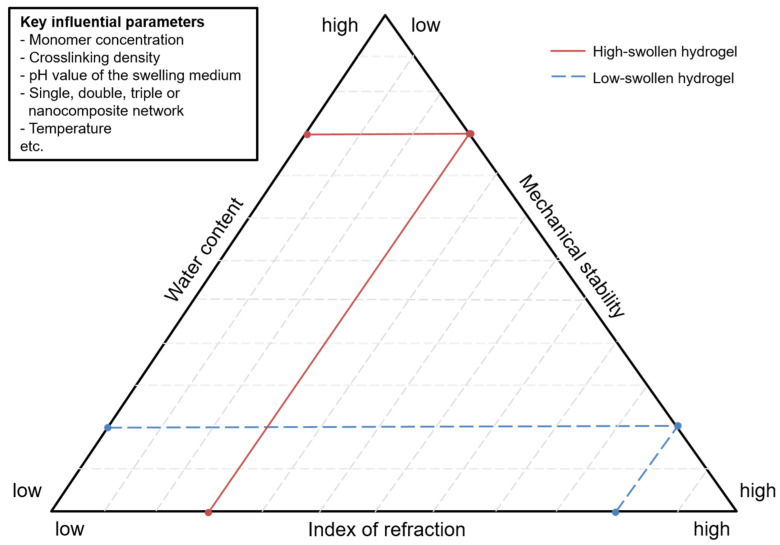
Triangular diagram to visualize the relation among the water content (swelling), mechanical stability, and IOR of the high- and low-swollen hydrogels with the key influential parameters of monomer concentration; cross-linking density; pH value; single, double, triple, or nanocomposite networks; and temperature.

The connection among the water content (swelling), IOR, and mechanical stability of the selected hydrogels in this review is illustrated by a triangular diagram shown in Figure 7. The three triangle sides represent the individual properties from low to high.

Two examples of swollen hydrogels are drawn in Figure 7. First, the continuous line represents a hydrogel with a high amount of water, leading to low mechanical stability and a low IOR. Second, the dotted line demonstrates a hydrogel containing lower water content with high mechanical stability and a high IOR.

The hydrogel PAMPS, for example, is an electrical-sensitive hydrogel. It showed deswelling kinetics under electric stimulation [32]. These properties can be used positively in PIV measurements. Especially in cardiovascular applications, blood flow in physiological and pathological vessels can be simulated in a PIV model. Thus, the mechanical properties of the PIV channel model could be adjusted during the test, and several research hypotheses could be investigated in one test setup.

## 7. Summary

Experimental cardiovascular flow simulations via PIV have been a trending topic for the last two decades. Current studies have shown substantial limitations. Materials such as silicon, PMMA, and resin have been utilized as PIV channel materials. This has made it almost impossible to simulate the natural pulsatile bloodstream in a vessel. Furthermore, there have been limitations in the matching of the IOR. By matching the fluid’s IOR to the one of the channel materials, the working fluid’s viscosity has changed. An evaluation of the relevance of IOR matching and viscosity is inevitable.

Two studies, by Oktamuliani et al. [65] and Shimizu and Ohta [66], presented hydrogels as a promising PIV channel material in cardiovascular research. Hydrogels can entirely replace standard materials such as silicon or PMMA. Physiological and pathological bloodstreams can be correctly simulated because of the hydrogels’ elastic properties. Another optical advantage is the high amount of water within swollen hydrogels, which makes them optically clear. The fluid’s viscosity remains untouched. Therefore, the IOR of the hydrogel can be perfectly matched to that of the aqueous fluid.

In current studies, only the hydrogel PVA has been utilized as PIV a channel material for cardiovascular simulations. This review presents seven other hydrogel groups that are suitable as channel materials, PAMPS, PAA, PVA, PAAm, PEG/PEO, PSA, and PNIPA. The hydrogel selection requirements were adapted to PIV measurements (optically) and the blood vessels to be simulated (mechanically). The optical properties were IOR and light transmission. The mechanical properties were the elastic modulus, tensile/compressive stress at break, nominal tensile/compressive strain at break, and water content. Table 3 lists all of these values for the selected hydrogels. These reviewed parameters are supposed to simplify the individual search for a suitable hydrogel as a PIV channel material. Here, the data are presented for cardiovascular research, but they can be used for other PIV application fields, such as turbine sciences.

In the future, this review should contribute to the increased use of hydrogels in PIV as a novel channel material. Research could benefit from the many advantages of synthetic hydrogels, such as high light transmission, elasticity, and mechanical properties.

## Figures and Tables

**Table 1:** Overview and description of hydrogels’ optical and mechanical measurement methods and their relevance for this review.

Property	Measurement Method	Description	Relevance to PIV Channel Material
***Optical***			
Index of refraction (IOR)	Refractometry [35,40,44,45,46,47]	Determination of the angle of refraction by the change in light direction in different materials	IOR matching between flow channel material and fluid
Infrared absorption	Fourier transform infrared spectroscopy (FTIR) [37,48,49,50,51,52]	Measuring the infrared absorption and emission spectra	Chemical hydrogel composition and structure
Raman scattering	Raman spectroscopy [48]	Measuring the inelastic scattering of monochromatic light on molecules or solids	Chemical hydrogel composition and structure
Light absorption	Ultraviolet and visible spectroscopy (UV/VIS) [38,39,53]	Light absorption in the visible and ultraviolet radiation range caused by electron transitions between different states in the molecule	Chemical hydrogel composition and structure; transparency of hydrogel
***Mechanical***			
Tensile/compressive stress	Universal testing machine (UTM) [37,48,49,52,53,54,55,56,57,58,59]	Determining the behavior of material samples under axial, tensile, or compression load	Mechanical durability and stiffness depending on hydration
Water vapor uptake and submission	Dynamic vapor sorption (DVS) [49,51,55,56,59,60]	Measuring material absorbability by varying the surrounding water vapor concentration	Hydrogel swelling and shrinking

**Table 2:** Mechanical properties (elastic modulus, tensile stress, and tensile strain) of healthy and atherosclerotic human coronary arteries [69].

	Elastic Modulus in MPa	Tensile Stress in MPa	Tensile Strain in %
Physiological	0.85–1.75	0.51–3.08	28–91
Pathological	3.13–4.27	1.11–3.59	27–60

**Table 3:** Mechanical and optical values for the seven hydrogel groups.

	Elastic Modulus in MPa	Tensile (*)/Compressive Stress at Break in MPa	Nominal Tensile (*)/Compressive Strain at Break in %	Water Content in wt.%	Index of Refraction	Light Transmission in %	Ref.
**Poly-2-acrylamido-2-methyl-1-propanesulfonic acid (PAMPS)**							
PAMPS/PAAm PAMPS/PAAm PAMPS/PAAm + silica nano- particle PAMPS/PAAm/PAMPS (cross-linked) PAMPS/PAAm/PAMPS (non-cross-linked) PAMPS/PAMPS PAMPS/PAA PAMPS/PTFEA PAMPS/PTFEA/PAAm PAMPS/MBAm + laponite PAMPS/PAAm	0.84 - 0.06–0.33 2 2.1 - - - - 0.69 -	4.6 17.2 18.6–73.5 4.8 9.2 3 2.3 1.6 * 21 27 -	65 92 94–97 57 70 80 75 4.9 * 97 - -	84.8 90 - 82.5 84.8 93 92 52 93 - -	- - - - - - - - - - 1.346–1.350	- - - - - - - - - - - -	[90] [49] [37] [90] [90] [49] [49] [49] [49] [54] [91]
**Polyacrylic acid (PAA)**							
PAA/PAAm PAA/alginate PAA/alginate + silica nano- particles PAA + sodium silicate	- - - 0.0128–0.0456	2.1 1.32 7.72–9.73 -	95 82.81 47.63–75.33 -	89 98.5 98.1–98.2 99.1–99.8	- - - -	- - - -	[49] [89] [89] [86]
PAA PAA/PEGMA + nanotitania hybrid film	- -	- -	- -	- -	1.527 1.501–1.528	- -	[92] [50]
**Polyvinyl alcohol (PVA)**							
PVA	-	2.45 *	650 *	85	-	-	[93]
PVA	0.38–2.28 * and 8.99–14.84	2.23–4.47 *	207.8–317.4 *	78.4–86.5	-	-	[94]
PVA + saline	0.7–18.4	1.4–2.1	45–62	75–80	-	-	[95]
PVA + nanocellulose	-	-	-	90.7–94.2	1.3330–1.3359	-	[44]
**Polyacrylamide (PAAm)**							
PAAm PAAm/PAAm PAAm/sPEOPO PAAm/PVA PAAm PAAm + sucrose PAAm PAAm/PAAm	0.63 * - 11.6–59.1 0.062–0.087 - - - -	1.1 * 5.4 2.0–5.6 - - - - -	81 * 92 88.6–93.2 469–500 * - - - -	- 92 92.3–95.2 - 89.8 - 75–95 92.23	- - - - - 1.385–1.420 1.338–1.380 1.343	- - - 92 98.2–98.9 - - -	[96] [49] [87] [38] [39] [36] [68] [40]
**Polyethylene glycol (PEG) and oxide (PEO)**							
PEG/PAA PEG/PAA PEG/PAA PEG-DA/PAA PEG-DA/MPEG PEO PEO/PEG	0.5–1.5 * - - - - - -	2–13 * 2.5–10.9 1.1 * 8 - - -	- 93.8–97.2 - 90 - - -	83–99 90 85 - 50–95 80–95 -	1.35 - 1.35 - 1.3388–1.4136 1.339–1.356 1.4539/1.459	90 - 96 - 97.6–100 - -	[45] [97] [46] [63] [68] [68] [92]
**Sodium polyacrylate (PSA)**							
PSA PSA/PAA/PBA PSA/PAAm	- - -	0.2–2.2 * 1.1–7.7 * -	5–115 * 1170–1730 * -	- - 80.79–99.02	- - 1.3327	- - -	[98] [88] [35]
**Poly-N-isopropyl acrylamide (PNIPA)**							
PNIPA + inorganic clay P(NIPA-co-AMPS)/PNIPA PNIPAm/PEGAAm PNIPA	0.4 * 0.085–0.311 4.10 -	1 * 2.532–17.50 0.175 * -	1000 * 71–95 56 * -	80–90 - 80 -	- - - 1.32–1.39	- - 90 -	[52] [99] [85] [53]

Value ranges were set as follows: elastic modulus-all values, tensile (*)/compressive stress at break-all values, nominal tensile (*)/compressive strain at break-all values, water content > 50 wt%, IOR < 1.55, light transmission > 90%. Mechanical terminology as adopted by ASTM D638-14 [70]. Abbreviations: polytetrafluorethylene (PTFEA), polyethylene glycol methacrylate (PEGMA), polyethylene oxide stat propylene oxide (sPEOPO), polyethylene glycol acrylamide (PEGAAm), methoxy polyethylene glycol (MPEG), diacrylate (DA), dimethacrylate (DMA), N,N’-methylenebis acrylamide (MBAm), 1,2-naph-thoquinone-2-diazide-5-sulfonic acid sodium salt (NQDSA), titan(IV) oxide (TiO_2_), polybutyl acrylate (PBA).
